# Cancer and HIV: The Molecular Mechanisms of the Deadly Duo

**DOI:** 10.3390/cancers16030546

**Published:** 2024-01-26

**Authors:** Aadilah Omar, Natasia Marques, Nicole Crawford

**Affiliations:** Division of Oncology, Department of Internal Medicine, Faculty of Health Sciences, University of the Witwatersrand, Johannesburg 2193, South Africa

**Keywords:** HIV/AIDS, viral load, cancer, antiretroviral therapy (ART), Kaposi sarcoma, invasive cervical cancer, non-Hodgkin’s lymphoma, AIDS-defining malignancy, non-AIDS-defining malignancy

## Abstract

**Simple Summary:**

Following infection with HIV, individuals are immunocompromised. Their weakened immune system puts them at a higher risk of certain cancers like Kaposi sarcoma and lymphoma. Even with antiretroviral therapy for HIV infection, these cancers remain common in developing countries, while in developed countries, there is a decline in some cancers but an increase in deaths from others. This review explores why these cancers happen in people with HIV and finds that the current treatment is not enough to prevent them. Understanding these reasons is crucial to improving the diagnosis and treatment of both HIV and its associated cancers, highlighting the need for more research to tackle this ongoing health issue.

**Abstract:**

The immune deficiency associated with human immunodeficiency virus (HIV) infection causes a distinct increased risk of developing certain cancer types. Kaposi sarcoma (KS), invasive cervical cancer and non-Hodgkin’s lymphoma (NHL) are the prominent malignancies that manifest as a result of opportunistic viral infections in patients with advanced HIV infection. Despite the implementation of antiretroviral therapy (ART), the prevalence of these acquired immunodeficiency syndrome (AIDS)-defining malignancies (ADMs) remains high in developing countries. In contrast, developed countries have experienced a steady decline in the occurrence of these cancer types. However, there has been an increased mortality rate attributed to non-ADMs. Here, we provide a review of the molecular mechanisms that are responsible for the development of ADMs and non-ADMs which occur in HIV-infected individuals. It is evident that ART alone is not sufficient to fully mitigate the potential for ADMs and non-ADMs in HIV-infected individuals. To enhance the diagnosis and treatment of both HIV and malignancies, a thorough comprehension of the mechanisms driving the development of such cancers is imperative.

## 1. Introduction

The immune deficiency associated with HIV infection causes a distinct increased risk of developing certain cancer types [1]. This phenomenon can be elucidated by predisposing factors such as immunosuppression in conjunction with persistent viral-induced chronic inflammation [2,3]. The HIV-infected population is also more inclined to engage in behaviors that elevate cancer risk, such as excessive alcohol consumption and tobacco use, when compared to the general population [4]. Furthermore, they are susceptible to frequent co-infections with other cancer-associated viruses like Epstein–Barr virus (EBV), human herpesvirus 8 (HHV-8), also known as Kaposi sarcoma-associated herpesvirus (KSHV), human papillomavirus (HPV), and hepatitis B and C viruses (HBV, HCV), which are aggravated in the presence of a compromised immune system [3]. Consequently, this leads to a higher probability of cancer development. KS, invasive cervical cancer and NHL are the prominent malignancies that manifest as a result of opportunistic viral infections in patients with an advanced HIV infection [5].

The occurrence of cancer in HIV-infected people has decreased slightly following the introduction and subsequent successful roll-out of ART [6], particularly in developed countries [7]. Despite this, individuals with HIV have an overall increased likelihood of developing cancer in contrast to those without HIV [4]. Additionally, HIV-positive individuals tend to experience a more aggressive progression of cancer and exhibit lower survival rates when compared to uninfected individuals [8,9]. Among HIV-positive patients, approximately 10–20% of all fatalities can be attributed to cancer [10,11]. Therefore, despite receiving ART, patients remain at a higher risk of developing malignancies. Furthermore, although ART lowers the risk of other ADMs, the association between ART and cervical cancer remains largely unclear [12]. Studies have also highlighted the lack of literature on cancers related to HPV in individuals with HIV across numerous countries in Sub-Saharan Africa. Additionally, it has been stated that there is insufficient research in rural areas and limited involvement of men in these studies [13].

The widespread adoption of ART and combination antiretroviral therapy (cART) has resulted in significant decreases in morbidity and mortality related to HIV/AIDS [14]. Over the past two decades, ART regimens have not only become more effective and less toxic but also simpler in terms of pill burden and frequency, thereby promoting better adherence [15]. This progress has translated into enhanced survival rates for individuals living with HIV who receive ART. In many regions today, HIV/AIDS is widely regarded as a manageable chronic condition, with the life expectancy of HIV-infected individuals undergoing ART approaching that of the general population in certain settings [16].

In developed countries, the persistence of ADMs has been more successfully circumvented, as the high adoption and early implementation of ART has resulted in fewer ADM-related deaths [17]. This has afforded a greater longevity and a better quality of life for those who are infected. Nonetheless, the likelihood of developing any cancers that are generally associated with AIDS remains significantly heightened in individuals with HIV. Thanks to the efficacy of antiretroviral drugs, individuals living with HIV are experiencing extended lifespans. Consequently, age-related diseases, including various types of non-ADMs, are emerging as increasingly significant health concerns within this population [18]. Individuals who have HIV are experiencing a higher occurrence and death rate associated with a broad range of non-ADMs [19].

Despite the highly successful use of ART to combat HIV infection, ADMs and non-ADMs remain a huge threat to the health of those who are infected. It is clear that ART alone cannot completely mitigate the risk of ADMs and non-ADMs in HIV-infected individuals [4,20,21]. A clear understanding of the mechanisms responsible for the development of such malignancies must be achieved to allow for a more efficient diagnosis and effective treatment of both HIV and malignancies. There is a pressing need for the immediate implementation of measures to prevent, and treat, cancers in individuals living with HIV. Here, we provide a review of some of the molecular mechanisms that are responsible for the development of ADMs and non-ADMs that result from the direct effect of the HIV infection with indirect factors being considered as well.

## 2. AIDS-Defining Malignancies

### 2.1. Inflammatory Cytokines and Signaling in the Formation of KS

**Figure 1:**
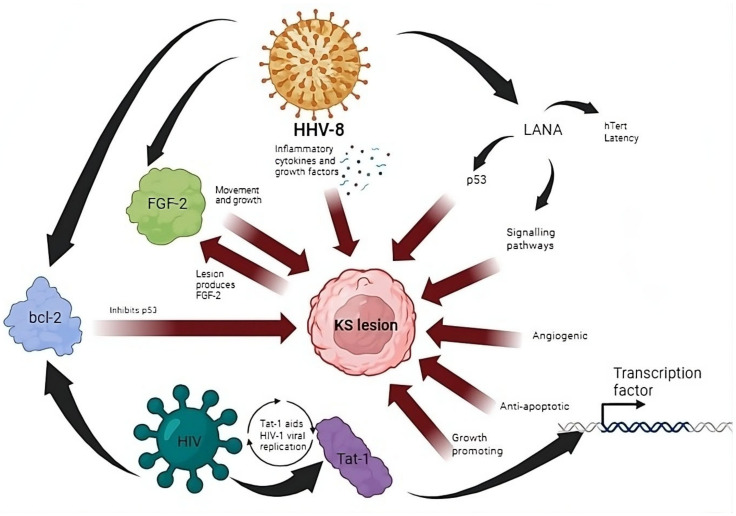
The various complex interactions between HHV-8 and HIV that lead to the formation of KS lesions. These include the production of inflammatory cytokines and growth factors by HHV-8. The lesion in turn produces FGF-2. The involvement of LANA leads to the inhibition of p53 and activation of signaling pathways. HIV in combination with HHV-8 causes bcl-2-mediated inhibition of p53. HIV Tat-1-1 protein acts a transcription factor to promote angiogenesis, growth and inhibition of apoptosis in addition to promoting viral replication.

One of the pertinent opportunistic diseases afflicting HIV-infected individuals is KS. As of 2020, the worldwide estimated age-standardized incidence rate (ASIR) for KS was 0.39 per 100,000 people, with approximately 34,270 newly diagnosed cases (23,413 males and 10,857 females). Africa represented a significant proportion, contributing to 73.0% of the global incidence and 86.6% of the total deaths from KS [22]. In HIV patients, KS progresses rapidly and aggressively with nodular lesions arising from an inflammatory granulation-type reaction [23,24]. One notable characteristic of KS development and sustainability is its intricate interplay within its microenvironment. This involves the production of inflammatory cytokines and signaling through paracrine mechanisms (Figure 1) [25]. The inflammatory mediators and growth factors that are responsible for eliciting the formation of these lesions are stimulated by HHV-8 [26]. This signaling includes factors like vascular endothelial growth factor (VEGF), platelet-derived growth factor (PDGF), fibroblast growth factor (FGF-2) and interleukin 6 (IL-6). These cytokines are associated with the progression and sustenance of KS [27]. An infection with both HHV-8 and HIV incites a cascade of complex events, with FGF-2, bcl-2 and Tat-1 being the key role players in these interactions (Figure 1). FGF-2 is a vital growth factor implicated in the progression of KS. Its expression by KS lesions or infiltrating leukocytes encourages movement and growth of endothelial and KS cells [23]. Additionally, certain proteins associated with HHV-8 inhibit p53-mediated apoptosis, including the viral bcl-2 protein, which acts as an apoptosis inhibitor [28]. The role of HIV-1 Tat-1 protein in cancer development will be discussed in more detail later in this review.

### 2.2. Oncogenic Viral Integration into Telomeres

Research has shown that various DNA and RNA oncoviruses have the capability of interacting with telomerase by integrating their genomic sequences into the telomeres of host cells [29]. Notably, certain herpesviruses, such as EBV [30] and HHV-8, have demonstrated the integration of their genomes into the telomeres of latently infected cells, leading to an upregulation of human telomerase reverse transcription (hTERT) promoter activity [31]. In HHV-8-related malignancies, most infected cells express the latency-associated nuclear antigen (LANA). LANA plays a crucial role in maintaining the viral episome DNA in dividing cells for an extended period and in upregulating the activity of the hTERT promoter [32] (Figure 1). Research indicates that the increased activity of the hTERT promoter is associated with LANA’s direct interaction with the transcription factor, Sp1 [33]. Moreover, LANA exerts regulatory control over the p53/Rb (retinoblastoma) and Wnt/β-catenin signaling pathways, thereby promoting the progression of B-cell lymphoma.

### 2.3. Immune Dysregulation and Immune Evasion in KS Development

The HHV-8 infection that is responsible for the immune response shown in Figure 1 establishes itself as a latent infection in transformed KS spindle cells. The propagation of HHV-8 is enhanced by HIV-induced immune dysregulation, resulting in immune responses that do not successfully curb viral infection but ironically aggravate the reactive process. In HIV-infected individuals, CD8+ T-cell activation and the production of Th1-type cytokines result in the formation of spindle-like cells and angiogenesis [23]. The crucial factor is the immune dysregulation that allows for HHV-8 infection advancement [24].

KSHV has evolved strategies to hinder the immune system at various stages, such as impeding viral detection, interfering with interferon signaling, suppressing cytokine production, and impairing immune cell recruitment and function [34]. The typical “cancer immunity cycle” process commences with the antigen priming of T lymphocytes, wherein dying tumor cells release antigens subsequently presented to the T-cell receptor. Following this, primed T-cells traverse from the lymph node and travel to the tumor site, where they identify and eliminate cancer cells [35]. Tumors employ immune-tolerant strategies through diverse mechanisms, including the manipulation of multiple co-inhibitory and co-stimulatory pathways. These include two critical immune checkpoints: cytotoxic T-lymphocyte-associated protein 4 (CTLA-4) and programmed cell death protein 1 (PD-1) pathways. The increased expression of PD-1 on cytotoxic T lymphocytes is a well-documented mechanism for immune tolerance in HIV-infected individuals [36]. Furthermore, in the context of HIV infection, prolonged inflammation can lead to immune exhaustion, resulting in a reduced ability to survey cancer and the creation of a favorable environment for oncogenesis [37]. Considering KSHV’s dependence on manipulating immune responses to effectively infect its host, immunotherapy emerges as an appealing approach for addressing KSHV-associated malignancies.

### 2.4. An Opportunity for Therapeutic Targeting

Traditional chemotherapy has been a long-standing treatment approach for KS, but numerous ongoing clinical trials are assessing the effectiveness of immune checkpoint inhibitors and anti-angiogenic agents in both HIV-associated and non-HIV-related KS [38,39]. Studies suggest that blocking PD-L1 can reverse immune dysfunction in HIV-specific CD8 T-cells, leading to improved survival, proliferation and cytokine production [40]. Additionally, Host et al. illustrated that KSHV infection in primary human monocytes increases the transcription and expression of PD-L1, revealing a novel immune evasion strategy for HHV-8 [41]. Since HHV-8 cannot be eliminated from the body, a complete cure for KS is not currently possible. However, long-term remissions are frequently observed, especially when addressing underlying immunosuppression. In cases of iatrogenic KS, altering immunosuppressive treatments, or in the case of HIV-associated KS, optimizing HIV infection control through cART should be prioritized as the initial preferred treatment where feasible [42].

It is important to highlight that there is currently no accepted animal model for Kaposi’s sarcoma (KS) or KSHV infection, hindering the testing of drug therapies before clinical trials. Consequently, in recent years, several targeted therapies for KS, particularly oral medications, have bypassed animal testing and directly entered human trials [36]. One particularly promising candidate is pomalidomide, an orally available derivative of thalidomide known for being less neurotoxic and exhibiting immune-modulatory, anti-angiogenic and anti-proliferative activities. In a phase 2 clinical trial, a total of 22 treated patients demonstrated an overall response rate of 73% [43]. Interestingly, a trial conducted using a comparable immunomodulatory agent, lenalidomide, yielded somewhat less favorable results, showing a response rate of 40% [44]. In the phase 2 trial evaluating the humanized monoclonal antibody bevacizumab, targeting VEGF-A in HIV-associated Kaposi’s sarcoma, the overall response rate was only 31% [45]. Imatinib, a selective inhibitor targeting the TKs Abl and c-kit, has undergone testing in HIV-associated Kaposi’s sarcoma, demonstrating promising results both in vitro and in vivo [46]. Rapamycin has proven efficacy in Kaposi’s sarcoma associated with transplantation and has demonstrated activity in AIDS-related Kaposi’s sarcoma. However, it is noteworthy that rapamycin exhibits significant drug–drug interactions with cART [47].

### 2.5. Cervical Cancer Formation Due to Co-Infection with HIV-1 and Multiple HPV Subtypes

Globally, the age-standardized incidence of cervical cancer was approximately 13.1 per 100,000 women, displaying considerable variation among countries, with rates ranging from under 2 to 75 per 100,000 women. Cervical cancer emerged as the primary cause of cancer-related mortality in women across East, West, Central and South Africa [48]. African nations still bear the highest burden of cervical cancer, primarily due to the high prevalence of HPV, HIV infection and inadequate coverage of cervical cancer screenings [49]. Research findings indicate that women with HIV face a 6-fold greater risk of developing cervical cancer in comparison to those without an HIV infection [50]. HIV significantly increases the risk of invasive cervical cancer, especially in Sub-Saharan Africa, where it contributes to a higher incidence of the disease [51]. It is highly prevalent in HIV patients and is commonly used as an indication of advanced HIV, or AIDS. Extensive research has concluded that persistent HPV infection is required for the formation of squamous intraepithelial lesions and is a chief contributor to the development of invasive cervical cancer [52,53,54]. Individuals with HIV, even with effective ART treatment, experience an elevated risk and rate of acquiring HPV, a more frequent presence of multiple HPV types and an increased incidence of HPV-related diseases, including a faster progression to malignancies [55]. In addition, since HIV and HPV are both sexually transmitted viruses, they share similar risk factors. This drastically increases the probability of acquiring both viruses. Furthermore, the diversity in HPV genotypes allows for a superinfection with multiple HPV strains, and studies have shown that co-infection with multiple HPV is associated with an excess of histologically confirmed cervical intraepithelial neoplasia grade 3 (CIN3) cases [56].

Studies have indicated that it is crucial to facilitate and initiate lifelong ART for individuals living with HIV, as this has been observed to positively influence the treatment response for cervical cancer. Further research is needed on the management of precancerous lesions and cervical cancer in HIV-seropositive patients, with a focus on the quality of life of those undergoing treatment. This research should also consider the effectiveness of the treatment method in relation to CD4+ count and the use of ART [57].

### 2.6. HPV Inhibition of Tumor Suppressor p53 as a Promising Therapeutic Strategy

Patients with HIV face an increased risk of developing cancers associated with HPV. This risk is only partially reduced by ART [58]. While these medications exhibit potency, conventional ART therapy often excludes protease inhibitors in current practice due to off-target effects observed during prolonged treatment. Notably, protease inhibitors, especially nelfinavir, have been explored beyond their role in HIV-1 treatment, being investigated independently as anticancer agents due to their ability to induce cell death in cancer cells [59]. Research has indicated that a minimum of four protease inhibitor drugs employed in the treatment of HIV/AIDS—lopinavir, ritonavir, nelfinavir and saquinavir—induce significant and specific reduction in HPV16 E6 and E7 oncoproteins in cancer cells positive for HPV [60]. HPV can induce the formation of neoplasia predominantly by disrupting the p53 tumor suppressor pathway [61,62]. HPV proteins, E6 and E7 in particular, are responsible for pathway dysfunction by deteriorating p53 function and promoting oncogene expression [63]. With the lack of cellular p53 control, apoptosis is averted, despite DNA damage [64]. E7 also affects the cell cycle regulator pRb, and in combination with the inactivation of p53 by E6, repeated uncontrolled cell division becomes apparent by the formation of warts. The involvement of these two oncoproteins in the dysregulation of the JAK/STAT-1 pathway has recently been explored making this signaling pathway a compelling target for therapeutic interventions [65]. In addition, the reactivation of endogenous p53 has the potential to enhance the sensitivity of cervical cancer (CC) cells to radiotherapy or chemotherapy (specifically, cisplatin + vincristine + bleomycin), as demonstrated in studies [66,67] Consequently, the reactivation of p53 has emerged as one of the most promising strategies for the effective treatment of cervical cancer in HIV patients [63,68].

### 2.7. Burkitt Lymphoma (BL) Caused by EBV Dysregulation of Protooncogene MYC

The World Health Organization (WHO) classifies BL into three clinical groups: endemic, sporadic and immunodeficiency-related [69]. The endemic form is linked to malaria and EBV. The immunodeficiency-related variant is associated with HIV, and to a lesser extent, organ transplantation. The predominant subtypes of HIV-associated NHL are diffuse large B-cell lymphoma (DLBCL) and Burkitt lymphoma (BL) [70,71]. BL stands out as a highly aggressive B-cell NHL characterized by the translocation and dysregulation of the proto-oncogene MYC. Beyond its initial descriptions, it has become evident that BL can manifest beyond the borders of Africa, affecting both pediatric and adult populations. In equatorial Africa, endemic BL is particularly prevalent, representing the most common pediatric malignancy in Sub-Saharan Africa [72]. This subtype often occurs with a 2:1 male predominance and emerges at a median age of 6 years [73]. Notably, endemic BL universally associates with EBV, indicating a likely direct role of the virus in the pathogenesis of this lymphoma [74]. EBV is capable of inducing lympho-proliferative disorders in immunocompromised individuals [75,76]. Patients living with HIV encounter a 10–20% lifetime risk of developing BL, irrespective of whether they receive ART [77]. In contrast to endemic BL, both sporadic and HIV-associated BL can affect individuals of all age groups. Despite variations in the clinical features and prognoses among the endemic, sporadic and HIV-associated forms [69,78], a common feature in all BL patients is the distinctive morphology and chromosomal translocation involving the MYC oncogene. This characteristic is present in BL cases regardless of geographical location or immunodeficiency status [79]. Another defining aspect of BL is its association with EBV infection. The endemic form is almost always EBV-positive, whereas less than 30% of sporadic BL tumors show EBV positivity [80]. BL constitutes nearly half of childhood NHL cases in Africa, and nearly all cases are linked to EBV [81,82].

### 2.8. NHL Development Due to Immunosuppression

The development of HIV-associated lymphoma involves multiple factors, some specific to HIV and distinct from the general pathogenesis of lymphoma. Elements include immunosuppression, immune dysregulation, the presence of HIV and co-infections with oncogenic viruses, particularly HHV-8 and EBV [83]. In terms of immunosuppression, findings from cohort studies indicate that higher levels of HIV viremia and a lower nadir of CD4 count are associated with an increased risk of developing lymphoma [84]. A study showed that within 2 years of an NHL diagnosis, 59% of patients infected with HIV succumbed to the disease, in contrast to 30% of patients not infected with HIV [85]. With the causative agent being of a viral nature, in combination with immune suppression being the key risk factor, the virus enters epithelial cells in the oropharynx, undergoes replication and then proceeds to infect B lymphocytes. Alternatively, it can directly infect B-cells in the tonsillar crypts. These B-cells circulate throughout the body and may experience either lytic infection, resulting in the production of a progeny virus, or more commonly, latent infection characterized by minimal viral gene expression. The link between EBV infection and lymphoma development in HIV patients could be utilized advantageously with accurate disease identification and prognosis being determined using CD4 counts in conjunction with EBV viral loads [86]. In individuals with HIV who are receiving cART, despite successfully managing viremia and enhancing CD4 T-cell counts, the risk of EBV-associated lymphoproliferative disorders (LPDs) remains elevated [87]. This persistent risk can be attributed to a combination of factors related to EBV-induced B-cell genetic transformation and a decrease in the effectiveness of immune surveillance [88].

### 2.9. Role of PTEN in BL Formation

Recent investigations into the function of the tumor suppressor gene PTEN in BL have revealed its role in impeding proliferation by downregulating p-AKT expression. It has been observed that PTEN-upregulated proapoptotic proteins, such as Bad and Bax, induce apoptosis. Additionally, it regulates cyclin-related factors, including P53, P21, CDK4, CDK6, cyclin D3 and cyclin H, to inhibit cell cycle progression. Furthermore, PTEN is involved in modulating epithelial–mesenchymal transition-like cell markers, including E-cadherin, N-cadherin, β-catenin, TCF-8, vimentin, Slug and Snail, thereby inhibiting cell migration and invasion. These findings underscore the significant role of PTEN in the development and pathogenesis of Burkitt lymphoma, offering valuable insights into its potential as a clinical target for treating this condition [89].

### 2.10. Immunomodulatory Approach Targeting EBV-Related Cancers

Initiatives aimed at advancing preventive and therapeutic strategies for EBV-LPD have investigated various approaches, including those directed at the virus and immunomodulatory interventions that target viral proteins [76]. These strategies involve adopting measures like the transfer of EBV-specific T-cells, which can be obtained from third-party donors or generated through stimulation with viral antigens [90]. Other avenues of exploration include the investigation of EBV-specific CD19 chimeric antigen receptor (CAR) T-cells. Notably, CAR T-cells designed to target the human leukocyte antigen-DR isotype (HLA-DR) have demonstrated successful elimination of EBV-transformed lymphoblastoid cell lines (EBV LCLs) in in vitro studies [91,92,93]. Another promising approach proven by positive outcomes in a phase 1 trial has been inducing a latent-to-lytic switch in the EBV infection state. Like for herpesvirus infections, these can be managed by inhibiting replication during the lytic phase, but their incurability arises from the establishment of latency [94]. Latency represents a dormant state characterized by the limited expression of genes. External factors can disrupt latency, leading to a burst of lytic reactivation that activates the entire viral genome. As this phase involves the expression of numerous viral proteins, making the virus susceptible to detection by the host immune system, the transition into the lytic cycle is rigorously regulated by a diverse array of mechanisms [95]. The latent-to-lytic switch is believed to enhance immune detection and destroy EBV-infected cells [96]. A deeper comprehension of the regulation of this life cycle could offer insights into disrupting it therapeutically. Lastly, an alternative and potentially valuable strategy might involve proactively vaccinating high-risk immunodeficient populations using a vaccine designed to induce a T-cell response. This vaccine would consist of an immunogenic antigen derived from EBV [97].

Immunocompromised patients can develop a remarkably varied range of EBV-associated lymphoproliferative disorders (LPDs) [79]. Recent advancements in prospective clinical trials contribute to establishing an evidence base for informed management. Additionally, emerging therapies like anti-CD30 antibodies, BTK and checkpoint pathway inhibitors, along with cellular therapies, offer potential for enhanced clinical outcomes in HIV-positive individuals [98]. Further research into the incidence of EBV LPD, mechanisms behind B-cell immortalization, various types of viral latency, and profiling of EBV-specific T-cells and natural killer (NK) cells in populations exposed to potential co-infections with HIV and EBV may provide additional insights into how viral immortalization and immune surveillance deficiencies interact, leading to an increased risk of virus-related cancers [91,99]. Continued investigation into the effects of immunomodulatory chemo-immunotherapy aimed at the biology of EBV-induced B-cell transformation and enhancing the immune response’s effectiveness is essential for optimizing the approach to treating EBV LPD in immunosuppressed populations, such as HIV-positive patients [76,100].

### 2.11. Overall View of ADMs in Africa

Significant strides have been conducted on the African continent in advancing our understanding of HIV-associated cancers. The continent has played a crucial role in contributing unique insights to the global knowledge base. Researchers and healthcare professionals in Africa have dedicated substantial efforts to unraveling the complexities of HIV-associated cancers, making invaluable contributions that extend beyond geographic boundaries [101]. These contributions encompass epidemiological insights, shedding light on the prevalence and distribution of these cancers in the region [102]. Additionally, African research has explored how unique HIV strains prevalent on the continent may interact differently with the immune system, influencing cancer development. The use of cancer biomarkers in assessing risk, diagnosis, prognosis and treatment for HIV-positive individuals is also being explored [103]. Importantly, these efforts have taken into account cultural and socioeconomic factors, providing a holistic understanding that shapes prevention and treatment strategies with cultural sensitivity [104]. African researchers have also tackled challenges specific to the continent, offering innovative solutions applicable on a global scale [105,106]. Moreover, collaborations between African researchers and the international scientific community have fostered a rich exchange of ideas and knowledge, enhancing research capacity within Africa and strengthening its ability to address the distinct healthcare challenges posed by HIV-associated cancers.

## 3. Post-ART Rise in Non-ADMs

Information from the US population-based HIV/AIDS Cancer Match (HACM) Study revealed a rise in the percentage of deaths linked to non-AIDS-defining cancers from 7.2% in to 11.8% in 2011–2015. However, the proportions of deaths attributed to AIDS-defining cancers remained steady at 5% throughout these time periods [107]. Early implementation of ART, routine screening and lack of childhood exposure to oncogenic viruses all contribute towards the reduced incidence and mortality associated with ADMs. This has been observed concurrently with a relative increase in the incidence of non-ADMs, which is believed to be linked to the suppression of HIV, bolstered immune responses and prolonged survival attributed to the development of new drugs [108]. There has been a shift towards the occurrence of non-ADMs, with an increase in the incidence of lung cancer, skin cancer, hepatocellular carcinoma, breast cancer, colorectal cancer, prostate cancer and Hodgkin’s lymphoma when compared to the pre-ART era [109,110,111]. Globally, non-AIDS-defining cancers are increasingly contributing to morbidity in individuals with HIV [112]. Studies on cancer disparities have shown a clear link between social determinants of health, such as insurance, healthcare access, education and income, with the likelihood adequate cancer screening [113]. Interestingly, studies have shown that such cancers occur more frequently in HIV-infected individuals than in the uninfected of a similar age, even though both groups are exposed to the same risk factors. A prolonged exposure to associated risk factors such as tobacco smoke, alcohol and UV radiation is inevitable with increasing age [114]. Moreover, the cancers that arise in HIV-infected individuals have been reported to present at a more advanced stage and show greater genetic instability than the same cancers found in HIV-uninfected individuals [110]. Based on these differences, it is probable that HIV infection is associated with promoting and supporting carcinogenesis.

Since the introduction of ART, the landscape of HIV/AIDS has evolved from an initially acute and terminal condition to a chronic illness. Analogous to many chronic diseases, it is associated with comorbidities. Despite some variations in specific rates or details among these studies, the overarching pattern of an increased cancer risk, surpassing that observed in the general population, poses a formidable challenge for those involved in the care of HIV-infected patients. Clinicians, researchers, policymakers, officials, patients and advocates are now compelled to address this challenge, akin to their concerted efforts in the early stages of the HIV epidemic, particularly regarding access to antiretroviral treatment and the subsequent management of AIDS-defining cancers. In the United States, a survey of radiation and medical oncologists revealed that nearly 20% were hesitant to provide standard-of-care cancer treatment to individuals living with HIV [115]. This reluctance was associated with the oncologists’ knowledge gaps regarding the safety and efficacy of treatments for this population. Subsequently, specific guidelines were established, recommending that individuals with HIV and good performance status should generally receive guideline-based cancer treatments. The guidelines also emphasize the importance of collaboration between oncologists, infectious disease physicians and pharmacists in managing potential drug interactions and side effects [116]. 

People living with HIV may face unique considerations influencing the benefits and risks of cancer screening, such as altered cancer risk, modified life expectancy, potential impacts on screening test performance and distinct outcomes from cancer therapies. Despite these considerations, there is a lack of HIV-specific cancer screening data and guidance. Guidelines suggest cancer screening appropriate for age and risk factors [117]. However, there is a need for future efforts to generate HIV-specific data for cancer screening, guiding the clinical implementation of established screening tools. In response to the escalating incidence of non-AIDS-defining cancers, there is a critical need to enhance early cancer detection, ensure the availability and utilization of effective treatments within the oncology community, and persistently work towards the ultimate goal of preventing these cancers and reversing this concerning trend in the years to come.

### 3.1. HIV-Related Chronic Inflammation in the Development of Cancers

**Figure 2:**
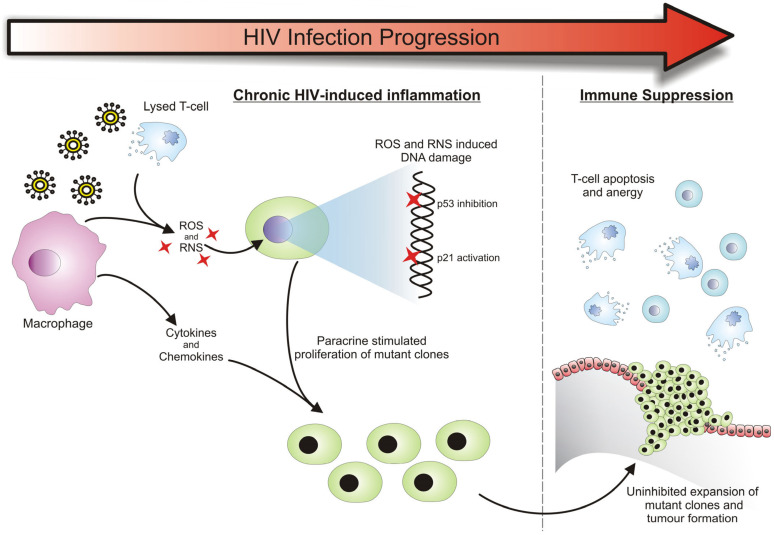
The continual exposure of the immune system to constantly changing HIV antigens and the resultant inflammatory stress has been implicated in the accelerated aging of the immune system, which is usually only seen at the end of a healthy individual’s lifetime. This enhanced “inflammaging” leads to the depletion of the naïve T-cell population and the accumulation of differentiated and anergic T-cells. These essentially ineffective HIV-targeting T-cells have a reduced capacity to proliferate and are susceptible to activation-induced apoptosis. These changes render the immune system ineffective in eliciting a strong immune response towards new antigens that might arise, compromising the immune surveillance and eradication of new cancerous cells. This includes chronic HIV-induced inflammation. Various inflammatory mediators produced during HIV infection, such as ROS and RNS, contribute to carcinogenesis by modifying the growth and survival of normal cells or triggering the proliferation of cells with tumorigenic potential.

Despite HIV viral replication being controlled by ART, the virus itself does activate the host’s immune system. This immune activation creates a state of chronic inflammation, which is strongly associated with neoplasia [111,118,119,120]. Various inflammatory mediators, which are produced as a result of the HIV infection, are able to induce carcinogenesis by altering the growth and survival of normal cells or triggering the proliferation of cells that already have tumorigenic potential. An example of this is the reactive oxygen and nitrogen species (ROS and RNS) that are produced in inflamed tissues to destroy invading pathogens (this process is described in Figure 2). During a state of chronic inflammation, there is an accumulation of such reactive species which can initiate the carcinogenic process. People with HIV infection demonstrate increased reactive oxygen species (ROS) generation in monocytes, along with significantly elevated concentrations of oxidized nucleic bases like 8-oxoG and lipid peroxidation products [121]. Large amounts of ROS and RNS can cause damage to cellular genomic DNA and cause genetic instability, potentially affecting the expression of genes that are key in regulating cell proliferation and survival [122]. The accumulation of such genetic alterations initiates the neoplastic process and is commonly seen in the development of cancers [123]. ROS and RNS are also capable of imparting post-translational chemical changes to proteins. Such changes often result in their aberrant activity, which can contribute to the carcinogenic process [118]. This was previously demonstrated by Calmels and Ohshima et al., who showed that nitric oxide (NO)-induced post-translational modifications to proteins have a transformative effect. In their study, such modifications resulted in the inhibition of the p53 tumor suppressor protein and the activation of the p21 proto-oncogene, which are both frequently observed perturbations in common cancers [124,125,126]. In addition to gene and protein damage, another consequence of chronic inflammation is the release of various cytokines, chemokines and growth factors that support the growth and proliferation of the newly and previously transformed cells [127] (Figure 1). Together, these factors provide an environment where genetic mutations can be acquired at an accelerated rate, giving the resultant clones the opportunity to expand and potentially dominate the area as a tumor.

The state of chronic inflammation, as a result of persistent HIV infection, eventually leads to the development of a contradictory state of immunosuppression. This allows for the continued progression of the tumorigenic process, with the growth and proliferation of transformed cells being uninhibited by the immune system [110,120]. Immune suppression occurs as a natural regulatory event in response to chronic inflammation [118]. However, it is further compounded by the decline in the function of the immune system—a phenomenon seen in HIV patients that is similar to the immunosenescence observed in the elderly [128]. The continual exposure of the immune system to constantly changing HIV antigens and the resultant inflammatory stress has been implicated in the accelerated aging of the immune system, which is usually only seen at the end of a healthy individual’s lifetime. This enhanced “inflammaging” leads to the depletion of the naïve T-cell population and the accumulation of differentiated and anergic T-cells [129]. These essentially ineffective HIV-targeting T-cells have a reduced capacity to proliferate and have short telomeres making them susceptible to activation-induced apoptosis [130]. These changes render the immune system ineffective in eliciting a strong immune response towards new antigens that might arise, compromising the immune surveillance and eradication of new cancerous cells.

Targeting cancer-promoting inflammation has emerged as a promising strategy, given the well-established evidence linking chronic inflammation to tumor progression and the suppression of immune anti-tumor responses. Various pharmacological agents designed to inhibit inflammatory cytokines or target macrophages within tumors have been developed and successfully tested in experimental tumor models. This success provides a strong rationale for clinical testing. Early clinical trials with these agents have shown that some patients experienced significant clinical benefits, including disease stabilization and prolonged progression-free survival, with manageable toxicity. These encouraging results are pivotal and set the stage for ongoing and future studies exploring combinations with conventional chemotherapy and biological therapies.

### 3.2. Cancer Susceptibility Due to a Deregulated Immune System

The deregulation of the immune system by a chronic HIV infection creates a tumor-enabling environment that can initiate carcinogenesis and support the process of tumor development. This phenomenon has been demonstrated in experiments performed on mice genetically engineered to lack various components of the immune system. It has been shown that such immunodeficient mice develop carcinogen-induced tumors much more frequently compared to the healthy controls [131]. Furthermore, an increased risk of developing cancers has been observed in organ transplant recipients, who are subjected to immunosuppression in order to prevent organ rejection [110,132]. A functional immune system is therefore imperative in suppressing transformed cells that have tumorigenic potential from developing into a tumor mass. This highlights the role of the chronic HIV infection in the development of non-ADMs and explains the increased risk of such individuals to develop such malignancies.

### 3.3. Cancer Formation Due to HIV Provirus Integration

Besides interfacing with and modulating the immune system, HIV also has direct contact with the host cell DNA during its life cycle. Some of these interactions have been described to have oncogenic potential. The HIV provirus has been previously thought to integrate randomly into the host genome [133]. This random integration is rather detrimental to the host as the function of important cell regulatory genes may be disrupted [134]. Other studies on the host flanking sequences of the provirus indicated viral integration is not random and occurs preferentially near transcriptional control genes [135,136]. A study conducted by Shiramizu et al. found that HIV-1 commonly integrates upstream of the c-fes/fps oncogene, resulting in its upregulation. This gene encodes for a protein tyrosine kinase that is involved in the transformation of hematopoietic cells. Other common integration sites of HIV-1 are those of the repetitive Alu elements and the intron of the BRCA 1 gene, which have both been implicated in breast, ovarian and colorectal cancers [133,137]. Upon integration into the host genome, HIV long terminal repeats (LTRs) exhibit functional capabilities that enable the amplification of the viral genome. HIV LTRs have been found to contain motifs that can serve as recognition sites for NF-κβ, cytokines and chemokines, which aid in its proliferation. A consequence of the recruitment of these factors is the upregulation of the genes downstream of the HIV integration site, such as that of the c-fes/fps oncogene. This was shown by an experimental construct that coupled the HIV 3′LTR upstream of the c-fes/fps gene, which resulted in a 10-fold increase in its expression in a human cell line [133,138]. Interestingly, when the viral at gene was added in cis to the construct, gene expression increased dramatically by a factor of 87-fold.

### 3.4. Role of HIV-1 Tat-1 Protein in Cancer Development

The Tat-1 gene product is known to play a significant role in HIV gene transcription and replication. As a consequence of its natural viral activity, transcription regulation studies have shown that the Tat-1 protein has the potential to alter host gene expression quite profoundly [139]. It has been found that Tat-1 has a pleiotropic effect in numerous cell types, displaying growth-promoting activity, angiogenic and anti-apoptotic functions [140], which all promote and support the hallmarks of cancer [126] (Figure 1). It achieves these functions by acting as a broad-range transcription factor to induce the expression of growth factors, cytokines, cytokine receptors and other transcription factors that aid in the replication of HIV. Tat-1 has also been found to be excreted from the cell into the extracellular milieu, where it interacts with cellular receptors to activate transduction pathways. These various activities of the HIV Tat-1 protein are suspected to be involved in neoplastic pathologies that arise in HIV-infected individuals, as many of its functions have an indirect tumor-promoting effect [139]. From this, it seems that the HIV Tat-1 protein contributes to the multistep development of cancer, by altering cellular functions that predispose cells to full neoplastic transformation, while aiding viral replication [141]. Studies have also revealed a correlation between the Tat-1 protein of HIV-1 and p53, linking it to the onset of cancers. The existence of Tat-1 nearly entirely hindered p53-mediated activation, resulting in diminished levels of p21 and the disruption of the G1/S checkpoint [142].

### 3.5. HIV-1 Nef Protein Involvement in Cancer

Lung cancer stands out as the most prevalent non-ADM and remains the primary cause of cancer-related mortality among individuals with HIV infection [143]. Notably, HIV-infected patients are diagnosed with lung cancer approximately ten years earlier than their uninfected counterparts [144]. Nef influences both HIV-infected and uninfected pulmonary vascular cells, as indicated by Ref. [145], and it is linked to substantial pulmonary vascular remodeling, along with lung lesions observed in humans, macaques and transgenic mice. The HIV-1 Nef protein is pivotal in AIDS progression and has been implicated in various oncogenic pathways [146,147]. It acts synergistically with vIL-6 to promote angiogenesis and tumorigenesis, facilitating the neoplastic transformation of immortalized neural cells. Furthermore, Nef hinders apoptosis, employing molecules like PI3K and PAK, playing a crucial role in inhibiting apoptosis and promoting cell survival during acute HIV-1 infection [148]. This function likely aims to prevent premature host cell death, allowing for viral replication and production [149]. The uptake of Nef protein by podocytes and pulmonary arterial endothelial cells in HIV-infected patients with nephrosis and pulmonary hypertension strongly indicates Nef’s role as a regulator of cell survival and proliferation. Studies also demonstrate the HIV-1 Nef protein’s involvement in altering the microenvironment of stromal and epithelial lung cells [150].

## 4. Conclusions

ART has revolutionized the prognosis of HIV infection, shifting it from a fatal condition to a chronic one. Paradoxically, this progress has led to cancer emerging as a leading cause of mortality among individuals with HIV. There are several mechanisms contributing to this phenomenon. Firstly, immunodeficiency resulting from HIV infection increases susceptibility to various cancers. Immunosuppression attributed to an HIV infection is the major contributing factor in the development of ADMs. KS and invasive cervical cancer occur as a result of an infection with the viruses HHV-8 and HPV, respectively, and in the context of a compromised immune system, the viral load is uncontrolled and often the consequence of this is neoplastic lesion formation. Additionally, HIV shares transmission routes with certain cancer-causing viruses, and the compromised immune system of people living with HIV (PLWH) makes them more vulnerable to viral infections. Viruses, in turn, can induce cancer through multiple pathways and the alteration of tumor-related gene expression. Recent evidence suggests that HIV itself directly contributes to the development of malignancies. Chronic inflammation, which is a hallmark of HIV infection, significantly promotes carcinogenesis. Interestingly, although immunosuppression is the major cause of malignancy development, HIV infection and its viral proteins play an important role in the mechanisms underlying cancer formation. This may be attributed to the action of specific viral proteins encoded by HIV, such as Tat-1 and Nef. Efforts are ongoing to identify inhibitors that target the pathways manipulated by HIV proteins, with promising results. Despite the successes in HIV viral load control seen following the implementation of ART, the incidence of ADMs remains high on the African continent. With time perhaps it will become possible for prompt ART delivery and adherence to therapy, which may cause a significant decrease in ADM incidence and mortality. On the other hand, even if ADM incidence is successfully curbed, those living with HIV are progressively more at risk of developing cancer with age. Taking into account the mechanisms by which ADMs as well as non-ADMs occur, it is imperative for the HIV viral load to be suppressed to maintain adequate immune system function. Moreover, routine screening for various common HIV-accompanying cancer types will allow for early detection and more effective therapeutic strategies.
